# Predictors of mortality in patients with severe sepsis admitted to an ICU

**DOI:** 10.1186/cc12946

**Published:** 2013-11-05

**Authors:** Fernanda Vilas Bôas Araújo, Fábio Ferreira Amorim, Adriell Ramalho Santana, Felipe Bozi Soares, Bárbara Magalhães Menezes, Jacqueline Lima de Souza, Mariana Pinheiro Barbosa de Araújo, Louise Cristhine de Carvalho Santos, Pedro Henrique Gomes Rocha, Lucas Garcia de Souza Godoy, Kátia Crys Moura Ogliari, Pedro Nery Ferreira Júnior, Alethea Patrícia Pontes Amorim, Rodrigo Santos Biondi, Rubens Antônio Bento Ribeiro

**Affiliations:** 1 Escola Superior de Ciências da Saúde, Brasília, Brazil; 2 Liga Acadêmica de Medicina Intensiva de Brasília, Brazil; 3 Hospital Anchieta, Brasília, Brazil

## Background

Severe sepsis is an important cause of morbidity and mortality for patients in ICUs [1]. Since instituting rapid treatment for patients with sepsis is critical, the need for reliable predictors of mortality to guide therapy is evident. This study attempts to identify the risk factors for mortality in patients admitted with severe sepsis to the ICU.

## Materials and methods

Case-control study conducted in the ICU of Hospital Anchieta, Brasília, DF, Brazil, during 5 months. Patients were divided into two groups: survivors group (SG) and nonsurvivors group (NSG).

## Results

During the study, 38 patients were admitted with severe sepsis, with a mortality rate of 47% (*n *= 18). Upon admission, the patients in the NSG presented higher values of: SAPS3 score (82 ± 12 vs. 60 ± 14, *P *= 0.00), heart rate (119 ± 21 vs. 99 ± 15 bpm, *P *= 0.00), serum creatinine (2.4 ± 1.4 vs. 1.5 ± 0.9 mg/dl, *P *= 0.00), decreased level of consciousness (92% vs. 58%, *P *= 0.03), need for vasopressor (85% vs. 25%, *P *= 0.00), need for invasive mechanical ventilation (62% vs. 12%, *P *= 0.00) and previous cardiac arrest (15% vs. 0%, *P *= 0.00). The platelet count was lower in the NSG (119,000 ± 70,000 vs. 220,000 ± 103.000/mm^3^, *P *= 0.00). There was no significant difference between the groups regarding the following factors: age (65 ± 19 vs. 65 ± 19 years, *P *= 0.98), respiratory rate (29 ± 9 vs. 26 ± 7 rpm, *P *= 0.30), axillary temperature (36.9 ± 0.7 vs. 37.2 ± 1.8°C, *P *= 0.43), leukocyte count (17,500 ± 7,800 vs. 13,000 ± 6.000/mm^3^, *P *= 0.08), immunosuppression (38.5% vs. 12.5%, *P *= 0.07), and prior use of corticosteroids (23% vs. 25%, *P *= 0.90).

## Conclusions

SAPS3 score, metastatic cancer, decreased level of consciousness, need for vasopressors, invasive mechanical ventilation, previous cardiac arrest, heart rate, serum creatinine, and platelet count were associated with mortality in severe sepsis for this sample of patients.
